# Clinical Performance of Direct Composite Restorations in Patients with Amelogenesis Imperfecta – Anterior Restorations

**DOI:** 10.3290/j.jad.b2838105

**Published:** 2022-03-24

**Authors:** Neslihan Tekçe, Mustafa Demirci, Safa Tuncer, Gizem Güder, Elif Ilgi Sancak

**Affiliations:** a Associate Professor, Department of Restorative Dentistry, Faculty of Dentistry, University of Kocaeli, Kocaeli, Turkey. Idea, placed the restorations, co-wrote the manuscript.; b Professor, Department of Restorative Dentistry, Faculty of Dentistry, University of Istanbul, Istanbul, Turkey. Hypothesis, experimental design, wrote the manuscript.; c Associate Professor, Department of Restorative Dentistry, Faculty of Dentistry, University of Istanbul, Istanbul, Turkey. Hypothesis, contributed substantially to discussion, proofread the manuscript.; d Dentist in Private Practice, Dent Design Dental Clinic, Sisli, Istanbul. Co-wrote the manuscript.; e Research Assistant, Department of Restorative Dentistry, Faculty of Dentistry, University of Kocaeli, Kocaeli, Turkey. Evaluated the restorations at recalls.

**Keywords:** amelogenesis imperfecta, dental enamel, composite resin, dental restoration, prospective study

## Abstract

**Purpose:**

To evaluate the clinical performance of direct composite restorations using nanohybrid and nanofill composite materials in anterior teeth in patients with amelogenesis imperfecta (AI).

**Materials and Methods:**

The study included 15 patients with AI aged 14–30 years. During the study, the patients received anterior direct composite laminate veneer restorations using either a nanohybrid (Clearfil Majesty ES-2 and Clearfil Universal Bond, Kuraray Noritake) or a nanofill resin composite (Filtek Ultimate Universal Restorative and Single Bond Universal Adhesive, 3M Oral Care). The restorations were evaluated according to the modified USPHS criteria at baseline and at 1-, 2-, 3- and 4-year follow-up periods.

**Results:**

The cumulative success rate of anterior restorations was 80.5% for nanohybrid and 92.5% for nanofill composite after 4 years. Eight restorations with nanohybrid and three restorations with nanofill resin composites failed. Ten restorations failed due to fracture; the fracture rate was 12.3%. Statistically significant differences were found between nanohybrid and nanofill composites regarding marginal discoloration and surface texture after 3 years. Furthermore, statistically significant differences were observed with respect to color match after 4 years.

**Conclusion:**

The use of a nanohybrid or nanofill composite for anterior direct restorations in patients with AI was observed to be satisfactory, based on the rate of ideal and clinically acceptable restorations. The primary reason for restoration failure was fracture. The failure rate of nanohybrid composite restorations was higher than with nanofill composite restorations with respect to survival and marginal adaptation criteria.

Amelogenesis imperfecta (AI) is a rare, inherited, and congenital disorder that primarily affects only enamel formation, without any associated morphological or metabolic defects. AI is predominantly classified based on clinical and radiographic evaluations of enamel defects as well as by the mode of its inheritance. Based on the clinical description, AI is classified into four types consisting of 14 subdivisions: hypoplastic AI, hypomaturation AI, hypocalcified AI, and hypomaturation-hypoplastic AI with taurodontism.^46^ The prevalence of AI varies from 1:700 to 1:14,000, according to the populations studied.^5^

Restorative treatment in patients with AI can be conducted through both direct and indirect treatment options.^29,37,41^ Direct restorations with resin-based composites are commonly preferred in young patients to avoid extensive preparation of teeth during adolescence.^41^ Also, they can be used in mild cases to veneer the surface of the teeth, or they can be utilized for more extensive buildups in more advanced cases.^37,41^ Indirect restorations can be used to restore teeth where extensive tooth tissue loss has occurred, and where moisture control is difficult to achieve for the direct buildup of teeth with a composite.^29^ Indirect treatment options include indirect composite restoration, post-and-core restorations, ceramic crowns, CAD-CAM restorations, porcelain veneers, and metal-ceramic fixed dental prostheses.^2,24,30,38,39,48^ Direct resin composites offer alternative treatment choices. Moreover, they provide excellent esthetics and a cost-effective restoration from both biological and economic points of view compared to other more invasive and expensive restorations.^29,37^ In addition, they ensure the preservation of tooth structure, given that the preparation is strictly limited to the areas of affected unsupported enamel.^37^ Therefore, composite resins should be considered before more invasive treatment options.^29^ Many case reports have addressed dental treatment of AI using direct composite restorations, revealing that direct composite restorations provide satisfactory esthetics and function in AI-affected teeth.^1,14,37,48,49^

Universal dental adhesives and nanohybrid and nanofill composites (nanocomposites) have been developed within the last few decades. They were designed for direct and indirect restorative approaches. Nanohybrid and nanofill composites show high translucency and high polishing properties, and their physical properties and wear resistance are equivalent to several hybrid and microhybrid composites.^7,25,42^ Furthermore, universal adhesives were designed under the all-in-one concept of existing one-step self-etch adhesives, but can also be used in different etch-and-rinse modes, such as etch-and-rinse and selective enamel etching.^19,31,43,45^ The addition of acidic functional monomers, such as 10-MDP, to universal adhesives, distinguishes them from the classic one-step self-etch adhesives.^31^ However, lack of data, particularly on the long-term clinical performance of universal adhesives, further complicates clinical decision-making.^27^

Our review of the current literature revealed no published studies that evaluated the clinical performance of anterior direct composite restorations in patients with AI. Therefore, the aim of this study was to evaluate and compare the clinical performance of direct composite restorations in patients with AI using nanohybrid and nanofill composite materials. The null hypothesis tested in this study was that there would be no significant difference between the clinical performance of the nanohybrid and nanofill composite restorative systems in anterior direct restorations of patients with AI after 4 years. 

## Materials and Methods

### Study Design

In this split-mouth, single-center, prospective clinical trial, patients received two different restorations with the two different composite materials under evaluation.^8^ The independent variables were the restorative material and time. Approval for the study was provided by the Ethics Committee of Kocaeli University, Faculty of Dentistry (KOU KAEK 2014/247). The patients were informed about the purpose of the study, treatment protocol, and study-related risks before beginning the study, and informed consent forms were signed by all patients or their guardians at the start of the study. The materials used are given in Table 1. This study included patients with AI who were enrolled for restorations between December 2014 and December 2016, in the departments of Restorative Dentistry, Faculty of Dentistry at Kocaeli University. Patients with AI who had been referred to the Department of Restorative Dentistry for treatment were examined by a practitioner (NT) who had experience with AI patients. In total, 15 patients (5 males and 10 females), with an age range of 14–30 years (mean: 19 years) were included in this study. The inclusion criteria were: clinically verified AI diagnosis, confirmed by anamnestic family history or clinical examination using Witkop’s classification, and treatment was necessary.^34,46^ The exclusion criteria were as follows: patients with developmental enamel defects of other origins – eg, fluorosis, molar incisor hypomineralization – and patients in whom AI was associated with systemic disorders and dental abnormalities such as open bite, deep bite, and cross bite.^23,34^ Oral hygiene and gingival health factors were recorded using Oral Health Progress Scoring (OHPR).^26^ Systemic diseases, allergies, pulpal diseases, and dietary habits were also taken into consideration. Each patient received two oral hygiene examinations per year using OHPR. This evaluation uses a simple criterion-based scoring for plaque, stain/calculus, gingival tissue (bleeding), and program acceptance. According to OHPR, “0” or “1” indicates excellent to good oral health, a score of “2” indicates borderline problems, and a score of “3” or higher signifies a definite problem in that area, requiring further evaluation or intervention with soft scaling of teeth for calculus, food impaction, or plaque.^26^ In the presence of scores of 2 and 3 at baseline, if a subsequent follow-up 2 weeks later indicated a successful intervention and excellent patient report, these patients were included in the study.^26^ Radiographs and photographs were taken of all the patients for diagnosis and treatment processing. The type of AI was diagnosed according to Witkop’s classification using photographs and radiographs to support clinical findings. Two other examiners (MD and ST) were subsequently included to evaluate the findings, and both examiners were given high-resolution images as reference instruments to confirm the initial diagnosis. Conflicts in diagnosis were resolved through consensus between the examiners. Following Witkop’s classification,^46^ of the 15 patients, 10 patients were diagnosed as having hypoplastic AI, four patients had hypomature AI, and one patient had snowcapped teeth of hypomature-type AI. The AI phenotype was determined based on the clinical presentation of the patient and radiographs.^13,29,36,46^ The clinical and radiographic characteristics of phenotypes of AI are shown in Table 2.

**Table 1:** Materials used in this study

Material Manufacturer	Ingredients	Application	Lot number
Clearfil Majesty ES-2 Nano-hybrid composite (Kuraray Noritake; Tokyo, Japan)	Organic content: bis-GMA, hydrophobic aromatic dimethacrylate, hydrophobic aliphatic dimethacrylate, dl-camphorquinone, accelerators, initiators Inorganic content: silanated barium glass filler, pre-polymerized organic filler. Inorganic filler: 78 wt%, 66 vol%, 0.37–1.5 μm	Place the chosen shade of the paste into the cavity and light cure with a dental curing unit. Considering the depth of cure, incremental curing may be required.	00020A 00006A
Filtek Ultimate Universal Restorative (Body) Nano-fill composite (3M Oral Care; St Paul, MN, USA)	Organic content: bis-GMA, UDMA, TEG-DMA, bis-EMA, PEG-DMA Inorganic content: a combination of non-agglomerated/non-aggregated 20-nm silica filler, non-agglomerated/non-aggregated 4- to 11-nm zirconia filler, and aggregated zirconia/silica, cluster filler (comprised of 20-nm silica and 4- to 11-nm zirconia particles). Inorganic filler: 72.5 wt%, 55.6 vol%, 0.6 μm–10 μm	Place and light cure restorative in increments for 10 s with Elipar S10.	N438989 N441522
Clearfil Universal Bond (Kuraray Noritake)	Bis-GMA, HEMA, 10-MDP, hydrophilic aliphatic dimethacrylate, colloidal silica, ethanol, dl-camphorquinone, silane, accelerators, initiators, water	Apply bond and rub it in for 10 s. Dry all cavity walls sufficiently with a mild air stream for more than 5 s. Light cure bonding agent with a light-curing unit.	2B0005
Single Bond Universal Adhesive (3M Oral Care)	10-MDP phosphate monomer, dimethacrylate resins, HEMA, Vitrebond copolymer, filler, ethanol, water, initiator, silane	Following selective enamel etching, apply the adhesive to the prepared tooth and rub it in for 20 s. Direct a gentle stream of air over the liquid for about 5 s. Light cure for 10 s.	494756

10-MDP: 10-methacryloyloxydecyl dihydrogen phosphate; bis-GMA: bisphenol A diglycidylmethacrylate; HEMA: 2-hydroxyethyl methacrylate.

**Table 2:** Clinical and radiographic characteristics of phenotypes of AI^13,29,36,46^

Phenotypes of AI	Clinical characteristics of AI	Radiographic characteristics of AI
Hypoplastic AI	Reduced enamel thickness Pitting and grooves in the enamel Hard and translucent enamel	The enamel contrasts normally from dentin.
Hypocalcified AI	Defects in enamel calcification Normal thickness enamel Weak structure of enamel Opaque or chalky enamel Teeth become stained and rapidly wear down	Enamel is less radio-opaque than dentin.
Hypomaturation AI	Enamel of normal thickness but with a mottled appearance with opaque white to yellow-brown or red-brown discolouration. Enamel is slightly softer than normal and vulnerable to tooth wear, but not as severe as the hypocalcified type.	Enamel has approximately the same radiodensity as dentin.
Hypomaturation-hypoplasia with taurodontism	Enamel is a mottled white-yellow-brown with pits most frequently on the labial surface or is thin with areas of hypomaturation.	Enamel has approximately the same or slightly greater radiodensity than dentin. Body and pulp chamber of molars enlarged, and the floor of pulp chamber and furcation is shifted apically down the root.

### Treatment Protocol

In the 15 patients, 46 direct laminate restorations were performed with a nanohybrid composite (Clearfil Majesty ES-2, Kuraray Noritake; Tokyo, Japan), and 45 laminate restorations were performed with a nanofill composite (Filtek Ultimate Universal Restorative, 3M Oral Care; St Paul, MN, USA). The nanohybrid composite was used with the proprietary universal adhesive (Clearfil Universal Bond, Kuraray Noritake) in selective etch mode (10 s etching time). The nanofill composite was also applied with a universal adhesive (Single Bond Universal, 3M Oral Care) in selective etch mode (15 s etching time). The present split-mouth study design compared the clinical performance of two different restorations using two different resin restorative systems by randomly allocating the restorations to half of each patient’s dentition, divided by the mid-sagittal plane, between the central incisors, as left and right sides of the dentition.^32,35^ Thus, restorations were started from the upper right, followed by the upper left, then the lower left and lower right quadrants of the mouth. The anterior teeth of the same-side quadrants received the same composite, creating a “split-mouth design.” A coin was flipped to determine which restoration would be made. Each restoration was made from the same materials. Thus, different restorations on the right and left sides of the mouth were symmetrically paired, except for one restoration pair. Table 3 shows the distribution of the restorations according to composite material and tooth number.

**Table 3:** Distribution of composite restorations according to composite material and tooth number

Materials	n	Tooth number											
		11	12	13	21	22	23	31	32	33	41	42	43
Nanohybrid composite	46	11	12	8	0	0	0	0	0	0	5	5	5
Nanofill composite	45	0	0	0	11	11	8	5	5	5	0	0	0

### Restorative Procedure

For operator calibration, direct laminate restorations were prepared on extracted anterior teeth using the materials tested in the study. Then, five direct laminate restorations per material were performed in patients without AI due to the very limited number of patients with AI. These restorations were not included in the study. First, the teeth underwent a cleaning process using a specially prepared pumice-water slurry, and then a rubber cup was used to remove the pellicle, as well as to remove any surface stains and any remaining residual dental plaque. After the teeth were cleaned, the shade was selected using the respective composite guide. The preparted cavities were moisture isolated using rubber-dam. The preparation also involved smoothing of surface irregularities and the removal of weakened, unsupported enamel, which in some cases contained little dentin. During the process, just the porous and colored enamel layer was removed. The average preparation depth was 0.5 mm, which remained within the enamel. The margins were not extended subgingivally. The preparation was extended just facial to the proximal contact point.^17^

Once the cavity preparation was complete, cavities were treated and the restorations placed strictly following the manufacturers’ instructions (Table 1). Cavity treatment, material application, and polymerization of dentin adhesives were conducted by the same experienced practitioner (N.T.), who had experience with the materials used in the study. The operator was blinded to the test materials. An Elipar S10 light-curing unit (3M Oral Care) was used for polymerization at an irradiance of 1200 mW/cm^2^. Then, the composite was placed in a single increment and light cured for 20 s. Finishing and polishing were performed during the same appointment. Subsequently, finishing was performed using micro-fine finishing diamonds. Finally, the restorations were polished using Sof-Lex abrasive disks (3M Oral Care).

### Evaluation

Two calibrated examiners with professional experience assessed the restorations under dental-unit lights using a dental explorer and a mirror, as per the modified United States Public Health Service (USPHS) criteria (Table 4).^3,10,15,22^ The examiners were not involved in the operation or the insertion of the restorations; thus, they were fully blinded to the experimental protocol. For training purposes, the examiners were provided with a set of pictures as a reference or as baseline instruments with which to compare each score for each criterion. Then, after leaving 2 days between each examination, the examiners clinically assessed 10 direct laminate veneers. Inter-examiner and intra-examiner agreement were tested using Cohen’s kappa coefficient. The assessment stage of the study was conducted only when the minimum threshold of 87% intra-examiner and inter-examiner agreement was attained in the calibration phase.^6^ During the baseline period, and subsequently at 1-, 2-, 3- and 4-year recalls, the properties of color match, wear and loss of anatomic form, marginal discoloration, caries, marginal adaptation, and surface texture were evaluated and scored (Table 5). During the scoring process, the following criteria were used: Alpha (A): ideal clinical findings; Bravo (B): clinically acceptable; Charlie (C); and Delta (D): clinically unacceptable, requires restoration replacement. Any conflicts during the scoring process were resolved through consensus.

**Table 4:** Direct clinical evaluation criteria (modified USPH criteria)

Rating	Aspect	Method
**Color match**		
Alpha (A)	No mismatch in color, shade and/or translucency between the restoration and the adjacent tooth structure.	Visual inspection
Bravo (B)	Mismatch in color, shade and/or translucency between restoration and adjacent tooth structure, but the mismatch is within the normal range of tooth color, shade and/or tranclucency.	Visual inspection
Charlie (C)	The mismatch is between restoration and adjacent tooth structure outside the normal range of tooth color, shade and/or translucency.	Visual inspection
**Cavosurface marginal discoloration**		
Alpha (A)	There is no discoloration anywhere on the margin between the restoration and the tooth structure.	Visual inspection
Bravo (B)	Discoloration anywhere on the margin between the restoration and the tooth structure, but the discoloration has not penetrated along the margin of the restorative material into enamel and can be polished away.	Visual inspection
Charlie (C)	The discoloration has penetrated along the margin of the restorative material into enamel.	Visual inspection
**Wear/anatomic form**		
Alpha (A)	The restoration is not undercontoured, that is, the restorative material is not discontinuous with existing anatomic form.	Visual inspection and explorer
Bravo (B)	The restoration is under-contoured, that is, the restorative material is discontinuous with existing anatomic form, but not enough restorative material is missing so as to expose the enamel or base.	Visual inspection and explorer
Charlie (C)	Enough restorative material is missing to expose the enamel or base.	Visual inspection
**Caries**		
Alpha (A)	There is no evidence of caries contiguous with the margin of the restoration.	Visual inspection
Bravo (B)	There is evidence of caries contiguous with the margin of the restoration.	Visual inspection
**Marginal adaptation**		
Alpha (A)	There is no visible evidence of a crevice along the margin into which the explorer will penetrate.	Visual inspection and explorer
Bravo (B)	There is visible evidence of a crevice along the margin into which the explorer will penetrate. The enamel or base is not exposed.	Visual inspection and explorer
Charlie (C)	There is visible evidence of a crevice along the margin into which the explorer will penetrate. The enamel or base is exposed.	Visual inspection and explorer
Delta (D)	The restoration is fractured or missing in part or in toto.	Visual inspection and explorer
**Surface texture**		
Alpha (A)	Surface of restoration is smooth.	Explorer
Bravo (B)	Surface of restoration is slightly rough or pitted, can be refinished.	Explorer
Charlie (C)	Surface deeply pitted, irregular grooves (not related to anatomy), cannot be refinished.	Explorer
Delta (D)	Surface is fractured or flaking.	Explorer

### Statistical Analysis

**Fig 1:**
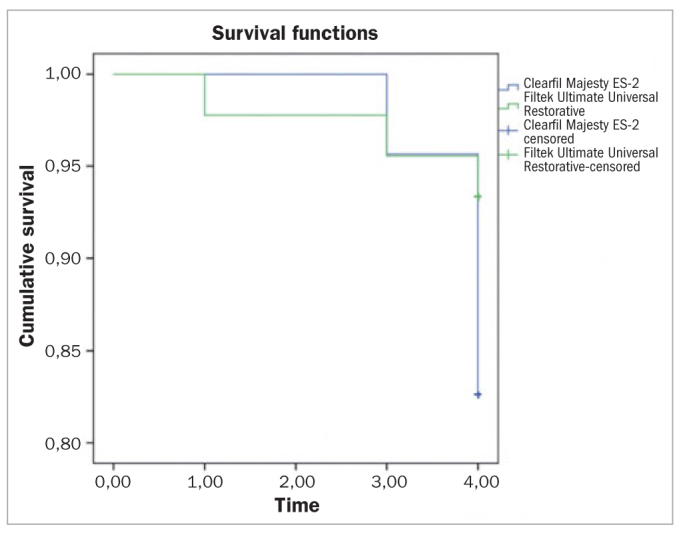
Kaplan-Meier survival analysis.

The SPSSWIN 20.0 (SPSS; Chicago, IL, USA) software was used for statistical analyses. Data related to the two resin composite restorative materials were analyzed statistically using the Friedman test for changes that happened throughout the 4-year evaluation period. The Mann-Whitney U-test was used to compare the materials at each time point for each evaluated criterion. When a statistically significant difference was identified for any criterion, Dunn’s post-hoc test was used for conducting multiple comparisons between each recall time interval for each composite resin material. Kaplan-Meier survival analysis was used to determine the probability of clinical survival of nanohybrid and nanofill composite resin (Fig 1). p-values <0.05 were considered statistically significant.

## Results

**Fig 2:**
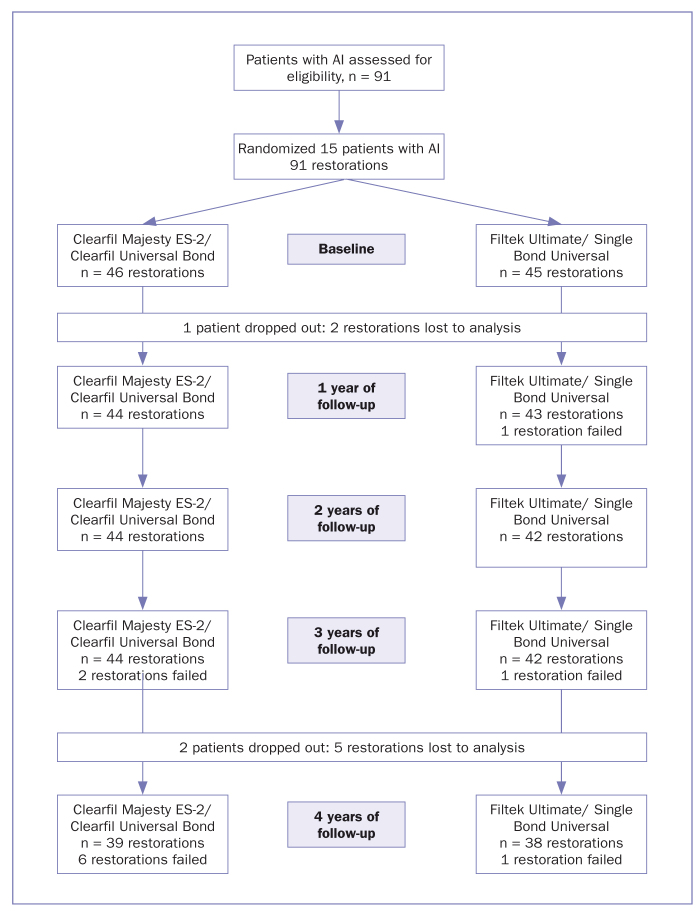
Flow diagram describing the history of the restorations.

During the four years of the study, two patients with 10 anterior restorations (5 nanohybrid composite restorations and 5 nanofill composite restorations) ceased participation in the study (Fig 2). This was because one patient replaced her/her restoration with prosthetic crowns after 1 year, and one patient moved to another city after 4 years. Therefore, the cumulative recall rate for the patients was 86.7% at the end of 4 years. After 4 years, 13 patients with 77 anterior teeth were left in the study. The cumulative recall rates at baseline and after 1, 2, 3, and 4 years are highlighted in Table 5. Cohen’s kappa coefficient (0.88) showed strong agreement between the examiners, with no statistically significant difference between them (p > 0.05).

**Table 5:** Results of clinical evaluation of direct composite restorations using modified USPHS criteria

Recall interval	Recall rate (number of restorations)	Retention		Color match			Marginal discoloration			Wear/anatomic form			Caries		Marginal adaptation				Surface texture			
		A	C	A	B	C	A	B	C	A	B	C	A	B	A	B	C	D	A	B	C	D
**Baseline**																						
Nanohybrid composite	100 (46)	100 (46) Aa		100 (46) Aa			100 (46) Aa			97.8 (45) Aa	2.2 (1)		100 (46) Aa		100 (46) Aa				100 (46) A			
Nanofill composite	100 (45)	100 (45) Aa		100 (45) Aa			100 (45) Aa			100 (45) Aa			100 (45) Aa		100 (45) Aa				97.8 (44) Aa	2.2 (1)		
**1 year**																						
Nanohybrid composite	100 (46)	100 (46) Aa		100 (46) Aa			100 (46) Aa			97.8 (45) Aa	2.2 (1)		100 (46) Aa		100 (46) Aa				100 (46) A			
Nanofill composite	100 (45)	97.8 (44) Aa	2.2 (1)	100 (44) Aa			100 (44) Aa			100 (44) Aa			100 (44) Aa		97.8 (44) Aa			2.2 (1)	95.5 (42) Aac	4.5 (2)		
**2 years**																						
Nanohybrid composite	93.3 (44)	100 (44)Aa		100 (44) Aa			97.7 (43) Aab	2.3 (1)		97.7 (43) Aa	2.3 (1)		100 (44) Aa		93.2 (41) Aab	6.8 (3)			100 (44) A			
Nanofill composite	93.3 (42)	97.7 (42) Aa	2.3 (1)	100 (42) Aa			100 (42) Aa			97.6 (41) Aa	2.4 (1)		100 (42) Aa		92.9 (39) Aab	7.1 (3)			92.9 (39) Aac	7.1 (3)		
**3 years**																						
Nanohybrid composite	93.3 (44)	95.5 (42) Aa	4.5 (2)	100 (42)Aa			90.5 (38) Ab	9.5 (4)		97.6 (41) Aa	2.4 (1)		100 (42) Aa		86.4 (38) Ab	9.1 (4)		4.5 (2)	100 (42) A			
Nanofill composite	93.3 (42)	95.3 (41) Aa	4.7 (2)	92.7 (38) Aa	7.3 (3)		100 (41) Ba			97.6 (40) Aa	2.4 (1)		100 (41) Aa		85.7 (36) Ab	11.9 (5)		2.4 (1)	87.8 (36) Bbc	12.2 (5)		
**4 years**																						
Nanohybrid composite	86.7 (39)	80.5 (33)Ab	19.5 (8)	97 (32) Aa	3 (1)		64.7 (22) Ac	32.4 (11)	2.9 (1)	100 (33) Aa			100 (33) Aa		65.8 (25) Ac	21.1 (8)		13.2 (5)	97 (32) A	3 (1)		
Nanofill composite	86.7 (38)	92.5 (37) Aa	7.5 (3)	70.3 (26) Bb	29.7 (11)		56.8 (21) Ab	43.2 (16)		94.6 (35) Aa	5.4 (2)		100 (37) Aa		76.3 (29) Ac	21.1 (8)		2.6 (1)	83.8 (31) Ab	16.2 (6)		

Observations are shown in % (cumulative number of restorations). A: Alpha; B: Bravo, C: Charlie; D: Delta. Different capital letters indicate significant difference between materials at recall time interval for each evaluation criterion. Different lowercase letters show statistically significant difference between each recall time interval for nanohybrid and nanofill composite for each evaluation criterion.

**Fig 3:**
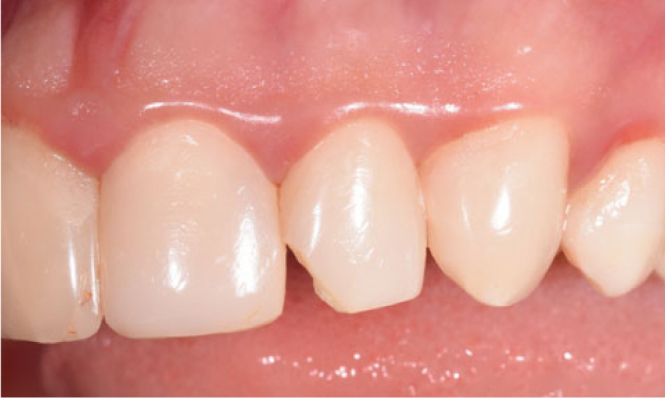
Clinical appearance after 3 years with failure (fracture).

**Fig 4:**
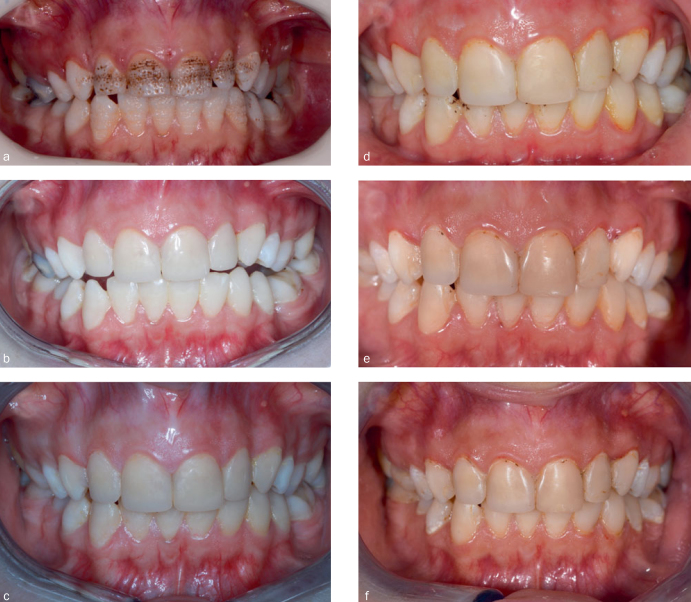
Intraoral view of composite laminate veneers on anterior teeth. a: baseline; b: 24 h; c: 1 year; d: 2 years; e: 3 years; f: 4 years.

The cumulative failure and success rates, according to Kaplan-Meier survival analysis, are shown in Fig 1 and Table 5 for anterior restorations. After 1 year, one nanofill composite restoration (2.2%) failed due to a fracture. There were no restoration failures at 2 years. At the end of the 3 years, two nanohybrid composite restorations (4.5%) and one nanofill composite restoration (2.4%) had failed due to a fracture (Fig 3). At 4 years, five anterior nanohybrid composite restorations (12.8%) and one anterior nanofill composite restoration (2.6%) fill had failed due to a fracture, and one nanohybrid composite restoration (2.6%) had failed due to unacceptable marginal discoloration. Thus, the cumulative success rate was 80.5% for nanohybrid composite restorations, and 92.5% for nanofill composite restorations at the end of the 4 years (Fig 4).

Statistically, a significant difference was found between the nanohybrid and nanofill composite restorations with respect to marginal discoloration (90.5% and 100%) (p = 0.044) and surface texture (100% and 87.8%) (p = 0.020) after 3 years, and color match (97% and 70.3%, respectively) (p = 0.003) after 4 years (Table 5).

## Discussion

The retention rate of restorations is the principal criterion for assessing the clinical effectiveness of adhesives, because, before a restoration is lost, its margins may have leaked extensively, undermining the restoration’s integrity without complete debonding. Although retention is the most objective criterion, ie, the restoration is still in place or debonded, clinical microleakage is far more difficult to evaluate objectively.^44^ The nanohybrid and nanofill composite restorations in this study showed acceptable clinical performance with 19.5% and 7.5% failure rates, respectively, for direct restorations in AI patients. Moreover, there was no significant difference between the success rates of nanohybrid and nanofill composite restorative systems based on retention rates. The hypothesis that there would be no significant difference between the clinical performance of nanohybrid and nanofill composite materials in anterior direct restorations of AI patients was also accepted. In this study, the 4-year retention rate of restorations for the nanohybrid composite was 80.5%, and for the nanofill composite 92.5%. The primary reason for restoration failure was due to restoration fracture. As opposed to our study, Chen et al^4^ found that 12 out of 23 direct anterior composite restorations had failed, and the failure rate of direct restorations was approximately 52% in the mixed dentition of 8 patients with AI. In a cross-sectional, retrospective study,^33^ it was found that the composite resin restorations had significantly shorter longevity in the AI group as compared with the control group. The present study did not include a “positive” control group regarding sound teeth with normal enamel. Therefore, it is very difficult to draw any conclusion about the extent to which enamel with AI affects clinical success compared with normal enamel. In our study, composite restorations were performed only in permanent teeth; also, most of the patients (10 patients) had hypoplastic type AI. The bond strength between enamel and adhesive restorative materials is highly dependent on the enamel surface modification.^4^ The structural alterations of AI-affected teeth may pose challenges to the bonding of adhesive restorations in clinical conditions.^12^ It has been shown that the enamel mineral content was reduced for all hypomaturation and hypocalcified AI teeth, and hypoplastic AI enamel varied from a normal to reduced state as compared with normal enamel.^46,47^

After 4 years, a statistically significant difference was found between restorations with nanohybrid and nanofill composites concerning their color match. Nanohybrid composite restorations more often had Alpha scores than did nanofill composite restorations in anterior teeth. In accordance with our findings, a 7-year retrospective analysis focusing on fractured maxillary teeth and diastema closure revealed that nanofill restorations had a higher rate of discoloration than did microhybrid restorations.^21^ Furthermore, an in vitro study showed that a microhybrid composite exhibited the least color change during the consumption of beverages, including a carbonated drink, tea, and distilled water, after 7 and 30 days; it also had a more stable color when compared with a nanofill composite.^28^ In contrast to our results, one study found no significant difference between nanohybrid and nanofill composites with respect to color match in the direct composite buildup restorations after 4 years.^9^

In the present study, seven nanohybrid composite restorations and three nanofill composite restorations failed due to a fracture after 1, 3, and 4 years. Also, two nanofill composite restorations failed because of marginal discoloration. The restoration margins were in enamel, and bonding to AI-affected enamel is more difficult than to normal enamel.^11^ Marginal discoloration and detectable margins are the only clinically measurable signs of the marginal seal of direct restorations.^16^ Furthermore, no typical etching patterns were detected in five clinical types of AI, namely, pitted hypoplastic, smooth hypoplastic, X-linked (male and female), and hypomineralized AI. The reason for this is described as the result of the abnormal prism/enamel structure, etching time, or acid concentration.^40^ In a cross-sectional retrospective study, among the causes of restoration failure, the rate of loss or fracture of a tooth was 60% for hypoplastic AI and 69% for hypomaturation/hypomineralized AI.^33^ Another study revealed that four out of 23 (17%) direct restorations showed unacceptable margins in regard to marginal integrity in the mixed dentition of AI patients.^4^ The difference between these and our studies may have been caused by differences in the patient’s age, restoration numbers, restoration materials, restoration types, and the type of AI.

The enamel of hypomineralized-type AI may have normal thickness, but the enamel is rough and soft, and it wears rapidly. In hypomature AI, the enamel is of normal size, and it contacts the adjacent teeth, but has a mottled, brownish-yellow, soft appearance.^18,33,46^ The prism structure shows abnormalities, and the bonding pattern is inadequate.^18,33^ In contrast, the enamel of hypoplastic AI has normal quality, but differs in its thickness.^18,33,46^ Therefore, in our study, the high rate of hypoplastic AI may have contributed to a higher rate of ideal restorations (Alpha) regarding marginal adaptation and marginal discoloration compared to the studies mentioned above. In agreement with our findings, a study with 4-year follow-up of AI cases using a one-bottle etch-and-rinse adhesive in direct labial veneers found satisfactory results with respect to marginal discoloration and marginal adaptation.^48^ Partially in contrast to our findings, in a 5-year follow-up case report of AI, 3-step etch-and-rinse adhesive with direct anterior composite restorations did not demonstrate signs of marginal degradation or marginal discoloration.^14^

After 4 years, 100% of nanohybrid composite restorations and 94.6% of nanofill composite restorations had clinically ideal properties regarding their level of wear and their anatomic form. A 5-year follow-up AI case report revealed that composite restorations aged without macroscopic signs of excessive wear, except for the loss of glossy surfaces, as was routinely observed in cases of extensive composite restorations.^14^ Only after 3 years was a statistically significant difference found between restorations with nanohybrid and nanofill composite in terms of surface texture; 97% of nanohybrid composite restorations and 83.8% of nanofill composite restorations were found to be clinically ideal in terms of surface texture. In agreement with our findings, there was no significant difference between microhybrid, nanohybrid, or nanohybrid composites for direct composite buildup restorations.^9,21^ In agreement with the results of Lempel et al,^21^ the nanofill composite restorations in this study received a lower rate of Alpha scores for color stability and surface texture than did the nanohybrid composite restorations. Nanofill composites are used to ensure high polishing with superior gloss and smoothness.^20^ A systematic review reported no in vitro evidence to support the selection of nanocomposites over the microhybrid composites, based on their superior surface gloss or smoothness.^20^

In the present study, no composite restoration exhibited caries adjacent to the margins in anterior teeth. In agreement with our findings, a 15-year case study reported that 18 months after the last treatment in permanent dentition, all restorations were intact, with no recurrent caries.^11^ Also, studies that had cases with a 4-year follow-up demonstrated that the restorations were still in favorable clinical condition without caries.^48^ In contrast to our results, in a cross-sectional retrospective study, recurrent caries accounted for 11% of the failures in hypoplastic AI and 21% in hypomaturation/hypomineralized AI.^33^ In a study that evaluated restorative treatment outcomes in the mixed dentition of AI patients, one out of 23 (4%) restorations in incisors showed unacceptable results regarding caries.^4^

## Conclusion

In the current study, the clinical performance of a nanohybrid and a nanofill composite used for direct restorations in patients with AI was found to be satisfactory, based on the rate of ideal and clinically acceptable restorations. Nanohybrid composite restorations performed better than nanofill composite restorations in terms of marginal discoloration, color match, and surface texture. The primary reason for the failure of the restorations was fracture.

## Clinical relevance:

Anterior direct composite laminate veneer restorations made of a nanohybrid and a nanofill composite material in patients with AI showed acceptable clinical performance after 4 years of clinical use.
